# Omega-3 Polyunsaturated Fatty Acids and the Resolution of Inflammation: Novel Therapeutic Opportunities for Aortic Valve Stenosis?

**DOI:** 10.3389/fcell.2020.584128

**Published:** 2020-11-12

**Authors:** Gonzalo Artiach, Magnus Bäck

**Affiliations:** ^1^Department of Medicine, Karolinska Institutet, Stockholm, Sweden; ^2^Department of Cardiology, Karolinska University Hospital, Stockholm, Sweden

**Keywords:** aortic valve stenosis, omega-3 fatty acids, resolution of inflammation, resolvin E1, valvular heart disease

## Abstract

Inflammation is well-established in cardiovascular disease, including valvular heart disease. Inflammation is a key process in the fibrosis and calcification of the aortic valve leaflets, which ultimately clinically manifest as aortic valve stenosis characterized by valve dysfunction and cardiac obstruction. In the absence of pharmacological treatment, either surgical or transcatheter aortic valve replacement is currently the only available therapeutic strategy for patients with severe aortic valve stenosis. Omega-3 polyunsaturated fatty acids, which exert beneficial effects in several cardiovascular diseases, serve as the substrate for several bioactive lipid mediators that regulate inflammation. Recent findings point to the beneficial effects of omega-3 fatty acids in cardiac valves, being inversely associated with aortic valve calcification and contributing to the resolution of valvular inflammation by means of the pro-resolving mediator resolvin E1 and downstream signaling through its receptor ChemR23.

## Introduction

Aortic valve stenosis (AVS) is the most common cause of interventional treatment of the cardiac valves (Nkomo et al., 2006), affecting over 10% of the population above 80 years (Eveborn et al., 2013). AVS is characterized by fibrocalcific aortic valve leaflets with reduced valve opening (Bäck et al., 2013). When AVS becomes severe, significant cardiac outflow obstruction develops, culminating in heart failure and, if untreated, death. Currently, there is no pharmacological treatment for AVS, leaving aortic valve replacement as the only available and effective treatment option.

The aortic valve opens and closes in a coordinated movement, allowing the blood flow from the left ventricle toward the aorta (Bäck et al., 2013). AVS is characterized by an increased inflammation, fibrosis, and calcification of the aortic valve leaflets, which together alter the valvular function. Increased mechanical stress on the aortic valve leads to endothelial injury and subsequent activation (Bäck et al., 2013), which, together with inflammatory cell infiltration (New and Aikawa, 2011), lipids and lipoproteins (Smith et al., 2014; Mathieu et al., 2017), extracellular matrix (ECM) remodeling (Aikawa et al., 2007), and oxidative stress (Miller et al., 2010; Mercier et al., 2020), are the key elements for the initiation of the valvular interstitial cell (VIC) calcification characteristic of the disease.

AVS has been associated with inflammation in several studies, manifested systemically by increased levels of C-reactive protein (CRP) (Galante et al., 2001). Calcified regions of stenotic human aortic valves contain infiltration of inflammatory cells (Aikawa et al., 2007), increased levels of inflammatory mediators (Nagy et al., 2011), and decreased levels of anti-inflammatory mediators (Artiach et al., 2020a). The central role of inflammation in AVS has received further support from animal models of aortic valve disease, which have demonstrated an increased lipoprotein infiltration accompanied by endothelial damage, inflammatory cell infiltration, and calcification of the aortic valve (Weiss et al., 2013). Macrophages and other inflammatory cells increase pro-inflammatory cytokines and ECM-degrading enzymes (Aikawa et al., 2007) that may lead to aortic valve thickening and, as a consequence, impaired valvular function. The ECM remodeling as a consequence of proteases and alterations in ECM synthesis will induce the generation of nidus for dystrophic calcification, characteristic in the development of the disease (Rodriguez and Masters, 2009). At later stages, VICs undergo an osteogenic differentiation and contribute to a heterotopic ossification process.

## The Resolution of Inflammation

The chronic inflammation in AVS, characterized by lipids, lipoproteins, inflammatory cell infiltration, and pro-inflammatory mediators, resembles that observed in atherosclerotic lesions. Importantly, atherosclerotic inflammation is characterized by a failure in the resolution of inflammation (Bäck et al., 2019). Challenging the concept of inflammation as a process that originally was thought to be resolved in a passive manner, the resolution of inflammation has emerged as an active process of limiting inflammatory cell infiltration and favoring phagocytosis for the removal of debris and apoptotic cells from the site of inflammation (Serhan, 2014). Macrophages, predominantly of the M2 subtype, play a key role in these processes (Ariel and Serhan, 2012), but also structural cells actively participate in the resolution of inflammation (Carracedo et al., 2019).

A group of bioactive lipids derived from polyunsaturated fatty acids (PUFAs) and coined specialized pro-resolving mediators (SPMs) are crucial for the resolution of inflammation to take place (Serhan, 2014). In addition to omega-6 PUFA-derived lipoxins, omega-3 PUFAs serve as the substrate for several families of SPMs (Serhan, 2014). Specifically, the D-series resolvins, maresins, and protectins are derived from docosahexaenoic acid (DHA), whereas eicosapentaenoic acid (EPA) gives rise to the E-series resolvins (Serhan and Levy, 2018).

SPMs exert their effects through specific G protein-coupled receptors (Pirault and Back, 2018). For example, resolvin E1 (RvE1) signaling through its receptor ChemR23 limits leukocyte activation (Arita et al., 2005) and enhances phagocytosis in atherosclerosis (Laguna-Fernandez et al., 2018). In addition, RvE1 promotes the anti-inflammatory M2-type macrophage polarization in heart tissue and spleen, shifting the LPS-induced pro-inflammatory M1-type phenotype, observed by increased expression of the M2 macrophage markers arginase 1, CD206, CD163, and CD36 (Zhang et al., 2020).

Stimulating SPMs and their receptors have beneficial effects in several animal models of intimal hyperplasia (Artiach et al., 2018; Liu et al., 2018), atherosclerosis (Petri et al., 2017), and vascular calcification (Carracedo et al., 2018). Recently, studies on SPM pathways (Artiach et al., 2020a) and M2 macrophages (Karadimou et al., 2020) pointed toward a failure in the resolution of inflammation as part of the pathophysiological processes underlying the chronic inflammation in AVS.

SPMs derived from omega-3 PUFA in addition have an important implication from a therapeutic point of view, in terms of omega-3 PUFA supplementation as a potential means of resolving cardiovascular inflammation. Indeed, the levels of CRP in coronary artery disease increase with lower dietary intake of omega-3 PUFA, in particular at an omega-6-to-omega-3 PUFA ratio above 4:1 (Artiach et al., 2020a,b; Sut et al., 2020). This has been further supported by studies in healthy volunteers where a high omega-6-to-omega-3 PUFA ratio is associated with increased levels of inflammatory biomarkers and CRP (Kalogeropoulos et al., 2010). Furthermore, the REDUCE-IT trial recently showed that treatment with EPA ethyl ester formulation in elevated doses conferred a 25% relative risk reduction in major cardiovascular events, including cardiovascular death, non-fatal myocardial infarction, non-fatal stroke, coronary revascularization, and hospitalization for unstable angina (Bhatt et al., 2019). Whether these beneficial effects of EPA were a result of a modulation of inflammation or/and other cardiovascular risk factors has not been established (Innes and Calder, 2020).

In addition to dietary intake and pharmacological supplementation, the omega-3 and omega-6 PUFA levels are also enzymatically regulated. In particular, the elongation of the very long chain fatty acids protein (ELOVL) together with the fatty acid desaturase (FADS) 1 and 2 catalyze the Δ5 and Δ6 desaturase activities necessary for the conversion of linoleic acid to arachidonic acid (AA) and the conversion of alpha-linolenic acid into EPA and DHA (Plunde et al., 2020).

## Omega-3 PUFAs in AVS

Genome-wide association and Mendelian randomization studies have identified a genetic variant in the FADS1/2 loci associated with AVS (Yuan et al., 2019), where each copy of the minor-C allele reduced more than 10% the odds of the disease (Chen et al., 2020). Since this variant genotype has been shown to alter both omega-3 and omega-6 PUFA metabolism and to lower AA generation from linoleic acid (Sasaki et al., 2019; Chen et al., 2020), those findings provide a potential link between PUFA and AVS. This is further reinforced by the finding that higher systemic levels of AA furthermore are associated with aortic valve calcification (Chen et al., 2020). However, the local valve levels rather than systemic levels of PUFA may be decisive for their availabilities as substrates for the biosynthesis of lipid mediators. Therefore, it is of importance that the protective minor allele of the FADS1/2 variant, in addition, confers an increased valvular FADS desaturase activity in the omega-3 PUFA pathway, increasing the amount of DHA in the aortic valve (Plunde et al., 2020). The latter study also showed that DHA levels were lower in calcified compared with non-calcified regions of human stenotic valves, recently extended to an overall decrease in omega-3 PUFA with aortic valve calcification (Artiach et al., 2020a). In addition, subjects with lower valvular omega-3 PUFA index have faster AVS progression prior to aortic valve replacement (Artiach et al., 2020a). Taken together, these studies support the idea that AVS progression and valve calcification are tightly linked to PUFA metabolism in general and to omega-3 PUFA in particular.

## Lipid Mediators and Specialized Pro-Resolving Lipid Mediators in AVS

Several pro-inflammatory lipid mediators derived from the omega-6 PUFA metabolome are locally generated in aortic valves. The expression of PUFA-metabolizing enzymes in, and the release of lipid mediators from, human aortic valves and VICs is shown in Table 1. For example, an upregulation of the 5-lipoxygenase pathway in calcified compared with non-calcified aortic valve regions induces a local release of pro-inflammatory leukotriene and correlates with calcification and infiltration of pro-inflammatory cells such as macrophages and T-lymphocytes (Nagy et al., 2011; Kochtebane et al., 2013; Artiach et al., 2020a; Table 1). Furthermore, the cyclooxygenase (COX) 1 and 2 enzymes that are essential for the generation of prostaglandins and thromboxanes from AA are expressed in human aortic valves (Suzuki et al., 2014; Sakaue et al., 2020). Interestingly, COX2 is upregulated in human calcified valves, and its inhibition reduces aortic valve calcification in a klotho-deficient mouse model of cardiovascular calcification via downregulation of osteogenic gene induction (Wirrig et al., 2015). However, the COX2 inhibitor celecoxib has also been associated with increased VIC calcification and AVS (Bowler et al., 2019; Vaidya et al., 2020).

**TABLE 1:** Fatty acid metabolizing enzymes and lipid mediator in human aortic valves.

	**Source**	**Method**	**Result**
**Fatty acid metabolizing enzymes**			
Cyclooxygenase-1	Aortic valves	IHC	↑ In AS valves (Suzuki et al., 2014; Sakaue et al., 2020)
	VIC	qPCR	↑ In VIC from calcified valve tissue (Sakaue et al., 2020)
Cyclooxygenase-2	Aortic valves	IHC	↑ In AS valves (Suzuki et al., 2014; Wirrig et al., 2015)
5-Lipoxygenase	Aortic valves	qPCR, IHC	↑ In calcified valve tissue (Nagy et al., 2011; Suzuki et al., 2014)
	VIC	qPCR, ICC	↑ After treatment with hypomethylating agent (Nagy and Bäck, 2005)
FADS1 and FADS2	Aortic valves	Microarray	↑ In calcified valve tissue (Plunde et al., 2020)
**Lipid mediator release**			
PGE2	VIC	ELISA	↑ In VIC from stenotic valve tissue in response do LPS, S1P (Fernandez-Pisonero et al., 2014)
LTB4	Aortic valves	ELISA	↑ In AS valves, correlated to degree of calcification (Kochtebane et al., 2013)
	Aortic valves	LC-MS/MS	↑ In calcified valve tissue (Artiach et al., 2020a)
	VIC	ELISA	↑ After treatment with hypomethylating agent (Nagy and Bäck, 2005)
RvD3	Aortic valves	LC-MS/MS	↓ (Trend) in calcified valve tissue (Artiach et al., 2020a)
RvE1	Aortic valves	LC-MS/MS	↓ In calcified valve tissue (Artiach et al., 2020a)

**AS, aortic stenosis; FADS, fatty acid desaturase; ICC, immunocytochemistry; IHC, immunohistochemistry; LC-MS/MS, liquid chromatography tandem mass spectrometry; LTB4, leukotriene B4; LPS, lipopolysaccharide; PGE2, prostaglandin E2; qPCR, quantitative polymerase chain reaction; RvD3, resolvin D3; RvE1, resolvin E1; S1P, shingosine-1-phosphate; VIC, valvular interstitial cell.**

Omega-3 PUFA supplementation decreases the generation of AA-derived lipid mediators generated through the lipoxygenase and COX activities (Ishihara et al., 2019), hence decreasing the inflammatory response. In addition, cytochrome P450-generated epoxygenation products from omega-3 PUFAs may contribute to these anti-inflammatory effects (Ishihara et al., 2019). Preventing subsequent epoxide metabolism through the inhibition of soluble epoxide hydrolase has been shown to sustain the anti-inflammatory actions of the omega-3 PUFA epoxygenation pathway (Zhang et al., 2014). As stated above, lipid mediators, which are enzymatically synthesized from omega-3 PUFAs, may in addition act to actively stop the chronic inflammatory process locally in the aortic valve by promoting the resolution of the inflammation. Indeed, the omega-3 PUFA-derived SPMs RvE1 and RvD3 have been detected using targeted mass spectrometry lipidomic analysis of human valves. RvE1, which is derived from EPA, is furthermore lower in calcified compared with non-calcified regions of aortic valves coming from aortic valve replacement (Artiach et al., 2020a).

These findings indicate that the balance between pro-inflammatory lipid mediators derived from omega-6 PUFA and anti-inflammatory as well as pro-resolving lipid mediators derived from omega-3 PUFA may be decisive whether the PUFA metabolism acts to increase or resolve chronic valvular inflammation. Similar leukotriene-to-resolvin ratios have been proposed as biomarkers in atherosclerosis to detect non-resolving chronic inflammation (Fredman et al., 2016; Thul et al., 2017). The use of SPMs as biomarkers for cardiovascular disease, however, needs further investigation (Calder, 2020).

## Omega-3 PUFAs Decrease Aortic Valve Disease

Increased systemic omega-3 PUFA as a result of introducing the Fat-1 transgene to hyperlipidemic mice is also reflected in an EPA and DHA enrichment in the aortic valve. The increased systemic and valvular omega-3 PUFA manifested as a hemodynamically reduced cardiac obstruction on echocardiography as well as reduced aortic valve leaflet thickness and calcification on histology (Artiach et al., 2020a). These observations hence provided the proof of concept for beneficial effects of omega-3 PUFA in AVS. The murine mouse model of aortic valve disease used in the latter study is characterized by valvular macrophage infiltration (Tanaka et al., 2005), endothelial activation, calcification, and upregulation of calcification-related proteins (Aikawa et al., 2007). Consistent with a potential stimulation of the resolution of inflammation by means of omega-3 PUFA, mice expressing the Fat-1 transgene in addition exhibit a polarization of the infiltrated valvular macrophages toward an M2 type, observed by increased expression of arginase 1, and a trend towards increased CD206 (Artiach et al., 2020a).

**FIGURE 1:**
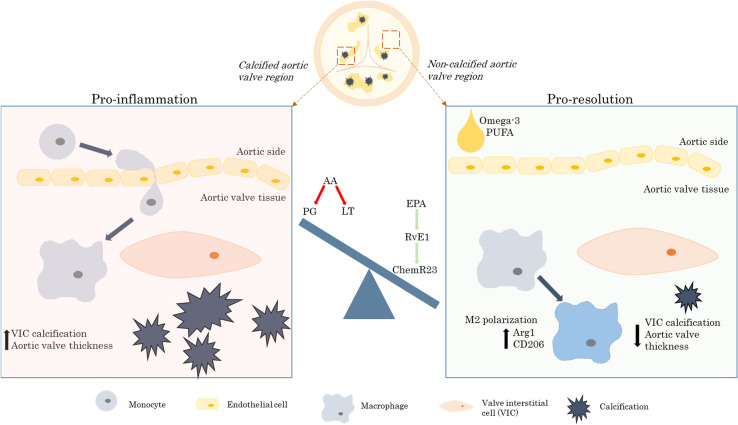
The beneficial effects of the omega-3 PUFA/RvE1/ChemR23 signaling axis in aortic valve stenosis. AA, arachidonic acid; Arg1, arginase 1; CD, cluster of differentiation; EPA, eicosapentaenoic acid; LT, leukotrienes; PG, prostaglandins; PUFA, polyunsaturated fatty acids; RvE1, resolvin E1; VIC, valvular interstitial cell.

Moreover, deletion of the RvE1 receptor ChemR23 exacerbated aortic valve disease and abolished the beneficial effects of omega-3 PUFA mediated by the transgenic Fat-1 expression, hence reinforcing the idea that the beneficial effects of omega-3 PUFA in aortic valve disease were mediated specifically by the RvE1 and ChemR23 signaling axis, as illustrated in Figure 1 (Artiach et al., 2020a).

## Molecular Mechanisms of Omega-3-Derived RvE1 in AVS

In support of RvE1 signaling affecting the valvular macrophage subtype, the mRNA levels of its receptor ChemR23 are significantly correlated with the expression of markers of M2-type macrophages in human stenotic valves (Artiach et al., 2020a). Similar associations have been reported also for Toll-like receptor (TLR) 7 in stenotic aortic valves and stimulation of macrophages with a TLR7 agonist induced release of the anti-inflammatory cytokine IL-10 (Karadimou et al., 2020), hence reinforcing the role of M2 macrophages in the resolution of valvular inflammation. Moreover, exogenous administration of RvE1 to cultured VIC reduced *in vitro* calcification, suggesting that omega-3 PUFA-derived SPMs in addition to altering the valvular immune response also directly act on the structural cells of the valve to counteract calcification. Similar findings are observed after RvE1 administration to vascular smooth muscle cells, where the reduced calcification by RvE1 is associated to lower expression levels of bone morphogenetic protein 2 (Carracedo et al., 2018).

The *in vivo* situation may however be more complex. ChemR23 has been identified as a transducer of opposing effects in different cell types in terms of, for example, blocking inflammatory activation of macrophages, leading to a reduced smooth muscle cell proliferation in a mouse model of intimal hyperplasia, but stimulating vascular smooth muscle cell proliferation *in vitro* when inflammatory cells are not present (Artiach et al., 2018). Furthermore, in contrast to the exacerbated aortic valve calcification after ChemR23 knockout in hyperlipidemic mice, a non-inflammation-dependent mouse model of vascular calcification induced by vitamin D3 displayed reduced vascular calcification after genetic deletion of ChemR23 (Carracedo et al., 2018). These observations hence indicate a close interplay between immune cells and structural cells in the cardiovascular system where the phenotype favored by SPM receptors may depend on the degree of inflammation (Carracedo et al., 2019).

## Discussion

Omega-3 PUFA exert a plethora of beneficial effects. One of the potential underlying mechanisms is by severing as substrate for SPM biosynthesis and mediating the resolution of inflammation (Serhan and Levy, 2018). Stimulating the resolution of inflammation to target chronic inflammation is an emerging concept as an alternative to anti-inflammatory treatment with the advantage to avoid immunosuppression. The applicability of the concept has been explored in atherosclerosis (Bäck et al., 2019) and is supported by recent clinical trials showing decreased cardiovascular risk by EPA ethyl ester treatment (Bhatt et al., 2019). Here, we provide arguments from recent studies that these beneficial effects of omega-3 PUFA extend to also AVS.

Increased systemic and valvular inflammation in patients with AVS may be a key pathophysiological process leading to increased calcification of the aortic valve and ultimately clinically manifested AVS. Omega-3 PUFA accumulates in both human and murine aortic valves (Artiach et al., 2020a; Plunde et al., 2020) and contributes to the formation of the SPM RvE1, which signals through its receptor ChemR23 to transduce the resolution of valvular inflammation. In this process RvE1-induced M2 macrophage polarization and direct anti-calcifying effects result in decreased hemodynamic and morphological signs of aortic valve disease (Artiach et al., 2020a).

Taken together, the preclinical and clinical findings on FADS/FADS 2 genotype, omega-3 PUFA, the EPA-derived SPM RvE1, and its receptor ChemR23 on aortic valve function and structure described in this review may open up novel clinical opportunities toward a medical therapy to prevent AVS and valvular calcification. In particular, the potential beneficial effects of omega-3 PUFA supplementation in AVS warrant further investigation in clinical trials.

## Author Contributions

GA and MB wrote the article. Both authors contributed to the article and approved the submitted version.

## Conflict of Interest

The authors declare that the research was conducted in the absence of any commercial or financial relationships that could be construed as a potential conflict of interest.

## References

[B1] Aikawa E. Nahrendorf M. Sosnovik D. Lok V. M. Jaffer F. A. Aikawa M. (2007). Multimodality molecular imaging identifies proteolytic and osteogenic activities in early aortic valve disease. *Circulation* 115 377–386. 10.1161/CIRCULATIONAHA.106.654913 17224478

[B2] Ariel A. Serhan C. N. (2012). New lives given by cell death: macrophage differentiation following their encounter with apoptotic leukocytes during the resolution of inflammation. *Front. Immunol.* 3:4. 10.3389/fimmu.2012.00004 22566890 PMC3342362

[B3] Arita M. Bianchini F. Aliberti J. Sher A. Chiang N. Hong S. (2005). Stereochemical assignment, antiinflammatory properties, and receptor for the omega-3 lipid mediator resolvin E1. *J. Exp. Med.* 201 713–722. 10.1084/jem.20042031 15753205 PMC2212834

[B4] Artiach G. Carracedo M. Clària J. Laguna-Fernandez A. Bäck M. (2018). Opposing effects on vascular smooth muscle cell proliferation and macrophage-induced inflammation reveal a protective role for the proresolving lipid mediator receptor Chemr23 in intimal hyperplasia. *Front. Pharmacol.* 9:1327. 10.3389/fphar.2018.01327 30515096 PMC6255922

[B5] Artiach G. Carracedo M. Plunde O. Wheelock C. E. Thul S. Sjovall P. (2020a). Omega-3 polyunsaturated fatty acids decrease aortic valve disease through the resolvin E1 and ChemR23 axis. *Circulation* 142 776–789. 10.1161/CIRCULATIONAHA.119.041868 32506925 PMC7439935

[B6] Artiach G. Sarajlic P. Bäck M. (2020b). Inflammation and its resolution in coronary artery disease: a tightrope walk between omega-6 and omega-3 polyunsaturated fatty acids. *Kardiol. Pol.* 78 93–95. 10.33963/KP.15202 32108752

[B7] Bäck M. Gasser T. C. Michel J. B. Caligiuri G. (2013). Biomechanical factors in the biology of aortic wall and aortic valve diseases. *Cardiovasc. Res.* 99 232–241. 10.1093/cvr/cvt040 23459103 PMC3695745

[B8] Bäck M. Yurdagul A. Tabas I. Öörni K. Kovanen P. T. (2019). Inflammation and its resolution in atherosclerosis: mediators and therapeutic opportunities. *Nat. Rev. Cardiol.* 16 389–406. 10.1038/s41569-019-0169-2 30846875 PMC6727648

[B9] Bhatt D. L. Steg P. G. Miller M. Brinton E. A. Jacobson T. A. Ketchum S. B. (2019). Cardiovascular risk reduction with icosapent ethyl for hypertriglyceridemia. *N. Engl. J. Med.* 380 11–22. 10.1056/NEJMoa1812792 30415628

[B10] Bowler M. A. Raddatz M. A. Johnson C. L. Lindman B. R. Merryman W. D. (2019). Celecoxib is associated with dystrophic calcification and aortic valve stenosis. *JACC Basic Transl. Sci.* 4 135–143. 10.1016/j.jacbts.2018.12.003 31061914 PMC6488810

[B11] Calder P. C. (2020). Eicosapentaenoic and docosahexaenoic acid derived specialised pro-resolving mediators: concentrations in humans and the effects of age, sex, disease and increased omega-3 fatty acid intake. *Biochimie* 178 105–123. 10.1016/j.biochi.2020.08.015 32860894

[B12] Carracedo M. Artiach G. Arnardottir H. Bäck M. (2019). The resolution of inflammation through omega-3 fatty acids in atherosclerosis, intimal hyperplasia, and vascular calcification. *Semin. Immunopathol.* 41 757–766. 10.1007/s00281-019-00767-y 31696250 PMC6881483

[B13] Carracedo M. Artiach G. Witasp A. Clària J. Carlström M. Laguna-Fernandez A. (2018). The G-protein coupled receptor ChemR23 determines smooth muscle cell phenotypic switching to enhance high phosphate-induced vascular calcification. *Cardiovasc. Res.* 115 1557–1566. 10.1093/cvr/cvy316 30597013

[B14] Chen H. Y. Cairns B. J. Small A. M. Burr H. A. Ambikkumar A. Martinsson A. (2020). Association of FADS1/2 locus variants and polyunsaturated fatty acids with aortic stenosis. *JAMA Cardiol.* 5 694–702. 10.1001/jamacardio.2020.0246 32186652 PMC7081150

[B15] Eveborn G. W. Schirmer H. Heggelund G. Lunde P. Rasmussen K. (2013). The evolving epidemiology of valvular aortic stenosis. *Tromsø. Study. Heart* 99 396–400. 10.1136/heartjnl-2012-302265 22942293

[B16] Fernandez-Pisonero I. Lopez J. Onecha E. Duenas A. I. Maeso P. Crespo M. S. (2014). Synergy between sphingosine 1-phosphate and lipopolysaccharide signaling promotes an inflammatory, angiogenic and osteogenic response in human aortic valve interstitial cells. *PLoS One* 9:e109081. 10.1371/journal.pone.0109081 25275309 PMC4183546

[B17] Fredman G. Hellmann J. Proto J. D. Kuriakose G. Colas R. A. Dorweiler B. (2016). An imbalance between specialized pro-resolving lipid mediators and pro-inflammatory leukotrienes promotes instability of atherosclerotic plaques. *Nat. Commun.* 7:12859. 10.1038/ncomms12859 27659679 PMC5036151

[B18] Galante A. Pietroiusti A. Vellini M. Piccolo P. Possati G. De Bonis M. (2001). C-reactive protein is increased in patients with degenerative aortic valvular stenosis. *J. Am. Coll. Cardiol.* 38 1078–1082. 10.1016/s0735-1097(01)01484-x 11583885

[B19] Innes J. K. Calder P. C. (2020). Marine omega-3 (N-3) fatty acids for cardiovascular health: an update for 2020. *Int. J. Mol. Sci.* 21:1362. 10.3390/ijms21041362 32085487 PMC7072971

[B20] Ishihara T. Yoshida M. Arita M. (2019). Omega-3 fatty acid-derived mediators that control inflammation and tissue homeostasis. *Int. Immunol.* 31 559–567. 10.1093/intimm/dxz001 30772915

[B21] Kalogeropoulos N. Panagiotakos D. B. Pitsavos C. Chrysohoou C. Rousinou G. Toutouza M. (2010). Unsaturated fatty acids are inversely associated and n-6/n-3 ratios are positively related to inflammation and coagulation markers in plasma of apparently healthy adults. *Clin. Chim. Acta.* 411 584–591. 10.1016/j.cca.2010.01.023 20097190

[B22] Karadimou G. Plunde O. Pawelzik S. C. Carracedo M. Eriksson P. Franco-Cereceda A. (2020). TLR7 Expression is Associated with M2 Macrophage Subset in Calcific Aortic Valve Stenosis. *Cells* 9:1710. 10.3390/cells9071710 32708790 PMC7407122

[B23] Kochtebane N. Passefort S. Choqueux C. Ainoun F. Achour L. Michel J. B. (2013). Release of leukotriene B4, transforming growth factor-beta1 and microparticles in relation to aortic valve calcification. *J. Heart Valve Dis.* 22 782–788. 24597398

[B24] Laguna-Fernandez A. Checa A. Carracedo M. Artiach G. Petri M. H. Baumgartner R. (2018). ERV1/ChemR23 signaling protects against atherosclerosis by modifying oxidized low-density lipoprotein uptake and phagocytosis in macrophages. *Circulation* 138 1693–1705. 10.1161/CIRCULATIONAHA.117.032801 29739755 PMC6200387

[B25] Liu G. Gong Y. Zhang R. Piao L. Li X. Liu Q. (2018). Resolvin E1 attenuates injury-induced vascular neointimal formation by inhibition of inflammatory responses and vascular smooth muscle cell migration. *FASEB J.* 32 5413–5425. 10.1096/fj.201800173R 29723062

[B26] Mathieu P. Arsenault B. J. Boulanger M. C. Bosse Y. Koschinsky M. L. (2017). Pathobiology of Lp(a) in calcific aortic valve disease. *Expert Rev. Cardiovasc. Ther.* 15 797–807. 10.1080/14779072.2017.1367286 28816078

[B27] Mercier N. Pawelzik S. C. Pirault J. Carracedo M. Persson O. Wollensack B. (2020). Semicarbazide-sensitive amine oxidase increases in calcific aortic valve stenosis and contributes to valvular interstitial cell calcification. *Oxid. Med. Cell Longev.* 2020:5197376. 10.1155/2020/5197376 32411328 PMC7201527

[B28] Miller J. D. Weiss R. M. Serrano K. M. Castaneda L. E. Brooks R. M. Zimmerman K. (2010). Evidence for active regulation of pro-osteogenic signaling in advanced aortic valve disease. *Arterioscler. Thromb. Vasc. Biol.* 30 2482–2486. 10.1161/ATVBAHA.110.211029 20864669 PMC2996870

[B29] Nagy E. Andersson D. C. Caidahl K. Eriksson M. J. Eriksson P. Franco-Cereceda A. (2011). Upregulation of the 5-lipoxygenase pathway in human aortic valves correlates with severity of stenosis and leads to leukotriene-induced effects on valvular myofibroblasts. *Circulation* 123 1316–1325. 10.1161/CIRCULATIONAHA.110.966846 21403093

[B30] Nagy E. Bäck M. (2005). Epigenetic regulation of 5-lipoxygenase in the phenotypic plasticity of valvular interstitial cells associated with aortic valve stenosis. *FEBS Lett.* 586 1325–1329. 10.1016/j.febslet.2012.03.039 22616993

[B31] New S. E. Aikawa E. (2011). Molecular imaging insights into early inflammatory stages of arterial and aortic valve calcification. *Circ. Res.* 108 1381–1391. 10.1161/CIRCRESAHA.110.234146 21617135 PMC3139950

[B32] Nkomo V. T. Gardin J. M. Skelton T. N. Gottdiener J. S. Scott C. G. Enriquez-Sarano M. (2006). Burden of valvular heart diseases: a population-based study. *Lancet* 36 1005–1011. 10.1016/S0140-6736(06)69208-8 16980116

[B33] Petri M. H. Laguna-Fernandez A. Arnardottir H. Wheelock C. E. Perretti M. Hansson G. K. (2017). Aspirin-triggered lipoxin A4 inhibits atherosclerosis progression in apolipoprotein E. *Br. J. Pharmacol.* 174 4043–4054. 10.1111/bph.13707 28071789 PMC5659998

[B34] Pirault J. Back M. (2018). Lipoxin and resolvin receptors transducing the resolution of inflammation in cardiovascular disease. *Front. Pharmacol.* 9:1273. 10.3389/fphar.2018.01273 30487747 PMC6247824

[B35] Plunde O. Larsson S. C. Artiach G. Thanassoulis G. Carracedo M. Franco-Cereceda A. (2020). FADS1 (Fatty Acid Desaturase 1) genotype associates with aortic valve FADS mRNA Expression, fatty acid content and calcification. *Circ. Genom. Precis. Med.* 13:e002710. 10.1161/CIRCGEN.119.002710 32397743 PMC7299231

[B36] Rodriguez K. J. Masters K. S. (2009). Regulation of valvular interstitial cell calcification by components of the extracellular matrix. *J. biomed. Mat. Res. A* 90 1043–1053. 10.1002/jbm.a.32187 18671262 PMC4230299

[B37] Sakaue T. Hamaguchi M. Aono J. Nakashiro K. I. Shikata F. Kawakami N. (2020). Valve interstitial cell-specific cyclooxygenase-1 associated with calcification of aortic valves. *Ann. Thorac. Surg.* 110 40–49. 10.1016/j.athoracsur.2019.09.085 31760051

[B38] Sasaki H. Sueyasu T. Tokuda H. Ito M. Kaneda Y. Rogi T. (2019). Aging and FADS1 polymorphisms decrease the biosynthetic capacity of long-chain PUFAs: a human trial using [U-13C] linoleic acid. *Prostaglandins Leukot. Essent. Fatty Acids* 148 1–8. 10.1016/j.plefa.2019.07.003 31492428

[B39] Serhan C. N. (2014). Pro-resolving lipid mediators are leads for resolution physiology. *Nature* 510 92–101. 10.1038/nature13479 24899309 PMC4263681

[B40] Serhan C. N. Levy B. D. (2018). Resolvins in inflammation: emergence of the pro-resolving superfamily of mediators. *J. Clin. Invest.* 128 2657–2669. 10.1172/JCI97943 29757195 PMC6025982

[B41] Smith J. G. Luk K. Schulz C. A. Engert J. C. Do R. Hindy G. (2014). Association of low-density lipoprotein cholesterol-related genetic variants with aortic valve calcium and incident aortic stenosis. *JAMA* 312 1764–1771. 10.1001/jama.2014.13959 25344734 PMC4280258

[B42] Sut A. Chiżyński K. Różalski M. Golański J. (2020). Dietary intake of omega fatty acids and polyphenols and its relationship with the levels of inflammatory markers in men with chronic coronary syndrome after percutaneous coronary intervention. *Kardiol. Pol.* 78 117–123. 10.33963/KP.15078 31790083

[B43] Suzuki K. Takahashi S. Watanabe K. Fujioka D. Nakamura T. Obata J. E. (2014). The expression of groups IIE and V phospholipase A2 is associated with an increased expression of osteogenic molecules in human calcified aortic valves. *J. Atheroscler. Thromb.* 21 1308–1325. 10.5551/jat.24273 25132377

[B44] Tanaka K. Sata M. Fukuda D. Suematsu Y. Motomura N. Takamoto S. (2005). Age-associated aortic stenosis in apolipoprotein E-deficient mice. *J. Am. Coll. Cardiol.* 46 134–141. 10.1016/j.jacc.2005.03.058 15992647

[B45] Thul S. Labat C. Temmar M. Benetos A. Bäck M. (2017). Low salivary resolvin D1 to leukotriene B. *Eur. J. Prev. Cardiol.* 24 903–906. 10.1177/2047487317694464 28195518

[B46] Vaidya K. A. Donnelly M. P. Gee T. W. Ibrahim Aibo M. A. Byers S. Butcher J. T. (2020). Induction of aortic valve calcification by celecoxib and its COX-2 independent derivatives is glucocorticoid-dependent. *Cardiovasc. Pathol.* 46:107194. 10.1016/j.carpath.2019.107194 31982687

[B47] Weiss R. M. Miller J. D. Heistad D. D. (2013). Fibrocalcific aortic valve disease: opportunity to understand disease mechanisms using mouse models. *Circ. Res.* 113 209–222. 10.1161/CIRCRESAHA.113.300153 23833295 PMC3786138

[B48] Wirrig E. E. Gomez M. V. Hinton R. B. Yutzey K. E. (2015). COX2 inhibition reduces aortic valve calcification in vivo. *Arterioscler. Thromb. Vasc. Biol.* 35 938–947. 10.1161/ATVBAHA.114.305159 25722432 PMC4979542

[B49] Yuan S. Bäck M. Bruzelius M. Mason A. M. Burgess S. Larsson S. (2019). Plasma phospholipid fatty acids, FADS1 and risk of 15 cardiovascular diseases: a mendelian randomisation study. *Nutrients* 11:3001. 10.3390/nu11123001 31817859 PMC6950527

[B50] Zhang G. Kodani S. Hammock B. D. (2014). Stabilized epoxygenated fatty acids regulate inflammation, pain, angiogenesis and cancer. *Prog. Lipid. Res.* 53 108–123. 10.1016/j.plipres.2013.11.003 24345640 PMC3914417

[B51] Zhang J. Wang M. Ye J. Liu J. Xu Y. Wang Z. (2020). The anti-inflammatory mediator resolvin e1 protects mice against lipopolysaccharide-induced heart injury. *Front. Pharmacol.* 11:203. 10.3389/fphar.2020.00203 32256344 PMC7094758

